# Biological Functions and Therapeutic Potential of NAD^+^ Metabolism in Gynecological Cancers

**DOI:** 10.3390/cancers16173085

**Published:** 2024-09-05

**Authors:** Subin Myong, Anh Quynh Nguyen, Sridevi Challa

**Affiliations:** 1 The University of Chicago Comprehensive Cancer Center, The University of Chicago, Chicago, IL 60637, USA; subin.myong@bsd.uchicago.edu; 2 Department of Obstetrics and Gynecology, The University of Chicago, Chicago, IL 60637, USA; anh.nguyen2@uchicagomedicine.org

**Keywords:** NAD^+^ (nicotinamide adenine dinucleotide), ADP-ribosylation, NAD^+^ metabolism, synthesis and consuming enzymes, NAD^+^ homeostasis, gynecologic cancers, metabolic regulation

## Abstract

**Simple Summary:**

NAD^+^ is a key cofactor that plays a role in various metabolic and signaling pathways. Understanding its ability to regulate cellular homeostasis also sheds light on its role in tumorigenesis and disease progression in gynecologic cancers. By reviewing the enzymes that consume NAD^+^ as well as the pathways and enzymes that synthesize NAD^+^, the vast influence NAD^+^ has on the survival of cancer cells becomes clear. With that information, advances have been made in the development of unique inhibitors to target NAD^+^ synthesizing and consuming enzymes.

**Abstract:**

Nicotinamide adenine dinucleotide (NAD^+^) is an important cofactor for both metabolic and signaling pathways, with the dysregulation of NAD^+^ levels acting as a driver for diseases such as neurodegeneration, cancers, and metabolic diseases. NAD^+^ plays an essential role in regulating the growth and progression of cancers by controlling important cellular processes including metabolism, transcription, and translation. NAD^+^ regulates several metabolic pathways such as glycolysis, the citric acid (TCA) cycle, oxidative phosphorylation, and fatty acid oxidation by acting as a cofactor for redox reactions. Additionally, NAD^+^ acts as a cofactor for ADP-ribosyl transferases and sirtuins, as well as regulating cellular ADP-ribosylation and deacetylation levels, respectively. The cleavage of NAD^+^ by CD38—an NAD^+^ hydrolase expressed on immune cells—produces the immunosuppressive metabolite adenosine. As a result, metabolizing and maintaining NAD^+^ levels remain crucial for the function of various cells found in the tumor microenvironment, hence its critical role in tissue homeostasis. The NAD^+^ levels in cells are maintained by a balance between NAD^+^ biosynthesis and consumption, with synthesis being controlled by the Preiss–Handler, de novo, and NAD^+^ salvage pathways. The primary source of NAD^+^ synthesis in a variety of cell types is directed by the expression of the enzymes central to the three biosynthesis pathways. In this review, we describe the role of NAD^+^ metabolism and its synthesizing and consuming enzymes’ control of cancer cell growth and immune responses in gynecologic cancers. Additionally, we review the ongoing efforts to therapeutically target the enzymes critical for NAD^+^ homeostasis in gynecologic cancers.

## 1. Introduction

NAD^+^ is a biomolecule integral to numerous essential biological processes. It is a key driver of energy production and a crucial signaling molecule, highlighting its significance in health and disease [1]. Cancer cells can reprogram their metabolism based on genetic, epigenetic, or microenvironmental changes, allowing increased growth rates and survival under limited energy resources and adverse conditions, thereby providing an advantage during tumor progression [2]. Along with its role in metabolic regulation, NAD^+^ acts as a key cofactor in various cellular signaling pathways through its ability to be consumed by enzymes such PARPs and sirtuins, giving it a key role in aging, the immune response, and various other pathways [3]. NAD^+^ signaling influences various processes that are dysregulated in cancer, including DNA repair, cell proliferation, differentiation, redox regulation, and oxidative stress [4]. Tumor cells exhibit higher ratios of the oxidized forms of NAD^+^ and NADP^+^ compared to nontumor cells, highlighting the crucial role of NAD^+^ in metabolic plasticity [5]. Tumor metabolism relies heavily on the pentose-phosphate pathway (PPP), serine biosynthesis, and fatty acid synthesis, all of which are dependent on NAD^+^ and NADP^+^. Thus, NAD^+^ biosynthesis becomes a key driver of cancer metabolism [6,7,8]. Furthermore, NAD^+^ is a vital regulator of cell signaling and survival pathways due to its role as a cofactor for enzymes essential in cellular energy production and various metabolic processes such as glycolysis, fatty acid oxidation, and the citric acid cycle [1]. NAD^+^ and NADH are crucial coenzymes in redox reactions, and an imbalance in their ratio can disrupt the flow of these pathways’ reactions, leading to a dysregulated cellular metabolism. Numerous studies have discovered that changes in NAD^+^ levels significantly contribute to metabolic disorders, neurodegenerative diseases, and tumorigenesis [5,9,10,11,12,13]. Here, we discuss the importance of cellular NAD^+^ metabolism in gynecologic cancer.

## 2. Regulators of NAD^+^ Homeostasis

### 2.1. NAD^+^ Biosynthesis

**Figure 1:**
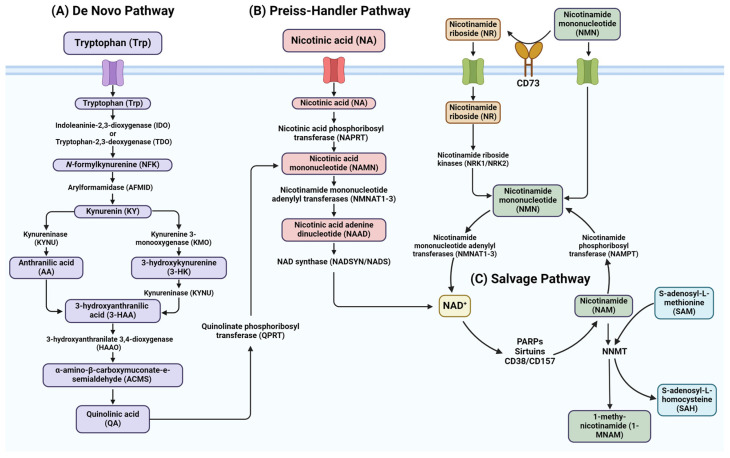
NAD^+^ biosynthesis pathways. NAD^+^ is synthesized through three different pathways, the de novo, Preiss–Handler, and salvage pathways. (**A**) The de novo pathway is the longest pathway, utilizing tryptophan (Trp) to produce quinolinic acid (QA) through a reaction cascade that feeds into the Preiss–Handler pathway. (**B**) The Preiss–Handler pathway converts nicotinic acid (NA) that has come from a dietary source into NAMN via NAPRT, which is then converted to NAAD by NMNAT enzymes allowing for the final step of forming NAD^+^ through NADSYN. (**C**) The NAD^+^ salvage pathway uses NAM, a product of enzymatic NAD^+^ consumption, back into NAD^+^ with NAMPT, regulating the rate limiting reaction in the process. Created with BioRender.com accessed 3 September 2024.

The NAD^+^ biosynthesis pathways are responsible for maintaining NAD^+^ levels within cells. There are three canonical pathways found in mammalian cells: the de novo synthesis pathway, the Preiss–Handler pathway, and the NAD^+^ salvage pathway [3] (Figure 1). As NAD^+^ is consumed, nicotinamide (NAM) is released, which can be recycled back through these pathways to replenish the NAD^+^ levels. Precursors for NAD^+^ can also be obtained from one’s diet in the forms of nicotinic acid (NA), nicotinamide (NAM), and nicotinamide riboside (NR), all of which are members of the vitamin B3 family [14]. This section will describe how these dietary precursors are used to synthesize NAD^+^ through the biosynthesis pathways.

#### 2.1.1. De Novo Pathways

The de novo synthesis pathway, or the kynurenine pathway, is responsible for breaking down the essential amino acid tryptophan into quinolinic acid (QA) through several enzymatically regulated steps. This pathway is one of the longest and most frequently active in organs such as the liver and kidneys [3]. The resulting quinolinic acid is converted to nicotinic acid mononucleotide (NAMN) by quinolinate phosphoribosyl transferase (QPRT), which then feeds into the Preiss–Handler pathway where the NMNAT enzymes use it to produce NAAD (Figure 1A) [15]. The rate-limiting step within this pathway is the conversion of tryptophan to its oxidized form *N*-formylkynurenine (NFK) by one of two enzymes: indoleaninie-2,3-dioxygenase (IDO) or tryptophan-2,3-deoxygenase (TDO). NFK is then converted to kynurenine (KYN) by arylformamidase (AFMID) [16]. The resulting KYN is then converted into either 3-hydroxykynurenine by kynurenine 3-monooxygenase (KMO) or anthranilic acid by kynureninase (KYNU), which becomes 3-hydroxyanthranilic acid (3-HAA). The enzyme 3-hydroxyanthranilate 3,4-dioxygenase (HAAO) then converts 3-HAA into α-amino-β-carboxymuconate-ε-semialdehyde (ACMS), which can spontaneously be catalyzed to form QA [17]. The pathway is also able to utilize aspartic acid as a precursor rather than tryptophan; though its main product is NAD^+^, the consumption of these amino acids in this pathway also helps to produce kynurenic acid, serotonin, and several other bioactive molecules [18].

#### 2.1.2. Preiss–Handler Pathway

The Preiss–Handler pathway highlights the intricate process by which dietary niacin and nicotinic acid (NA) are utilized to produce NAD^+^ (Figure 1B). The enzyme nicotinic acid phosphoribosyl transferase (NAPRT) is the rate-limiting step in this pathway, converting nicotinic acid to the intermediate nicotinic acid mononucleotide (NAMN). NAMN is then converted into nicotinic acid adenine dinucleotide (NAAD) by nicotinamide mononucleotide adenylyl transferases (NMNATs) [19]. Its final step then utilizes NAD^+^ synthase (NADSYN1 or NADS)-to catalyze them replacement of the carboxylic group in NAAD with an amide group from glutamine to produce NAD^+^ as its final product [20].

#### 2.1.3. NAD^+^ Salvage Pathway

When NAD^+^ is consumed by various enzymes such as PARPs, the resulting nicotinamide (NAM) serves as a precursor to resynthesize new NAD^+^ molecules through the salvage pathway. It is in this pathway that the reaction product or dietary nicotinamide mononucleotide (NMN), nicotinamide riboside (NR), and nicotinamide (NAM) can be converted to NAD^+^ (Figure 1C) [19]. In many mammalian systems, the rate limiting step of this process is mediated by the enzyme nicotinamide phosphoribosyl transferase (NAMPT) [10]. NAMPT plays the crucial intracellular role of mediating NAD^+^ homeostasis through regulation of the salvage pathway, but the molecule itself is not restricted to that single role. The enzyme is noted to have two forms, intracellular NAMPT (iNAMPT) and extracellular NAMPT (eNAMPT), with separate functions due to their localizations [21]. Though the mechanisms surrounding the function and secretion of eNAMPT are not fully understood, evidence suggests that eNAMPT is released as an inflammatory cytokine, making it a key immunity regulator [22,23].

Initially identified in 1994 as a cytokine secreted by pre-B cells and activated lymphocytes, eNAMPT was named “pre-B cell colony enhancing factor” (PBEF) for its role in enhancing pre-B-cell colony formation in synergy with interleukin (IL)-7 and various stem cell factors (SCFs) [24,25,26]. Additional studies have also found that eNAMPT can be carried through blood vessels in extracellular vesicles and is able to enhance NAD^+^ biosynthesis in hypothalamic neurons in mice and humans [27]. Moreover, Yoshida et al. was able to demonstrate the influence of eNAMPT in various types of tissues such as adipose tissue, pancreatic tissue, the retina, and the hippocampus by altering their function through changes in NAD^+^ levels [27].

Though iNAMPT constitutes the rate limiting step of the salvage pathway, it is not the final step of the pathway. The resulting NMN produced by NAMPT from NAM is converted to NAD^+^ by NMNAT enzymes [3]. Among the three human NMNAT isoforms, their differences are found in their distinct subcellular localizations, with NMNAT1 being distinctly found in the nucleus, NMNAT2 in the cytosol, and NMNAT3 in the mitochondria [28]. Because the NMNAT enzymes inhabit different areas of cells, their expression levels influence the levels of subcellular NAD^+^ levels and help regulate the functions of different cell types [29]. The expression levels of the NMNATs have been found to be heavily dependent on tissue type, with NMNAT1 being ubiquitously expressed in different tissues [30]. NMNAT2 has been found to be limited to the brain and pancreatic, heart, and skeletal muscle tissue, while NMNAT3 is greater expressed in the spleen, kidneys, and lungs [28]. Studies have shown that NMNAT2 plays a key role in axon maintenance and function, with the depletion or loss of active NMNAT2 causing neurite degeneration [11,12]. Since the salvage pathway is heavily influenced by the consumption of NAD^+^ in converting its substrates NAM, NMN, and NR (Figure 1C), changes to NAD^+^ levels can be influenced by either inhibiting the salvage pathway enzymes or promoting the activity of NAD^+^-consuming enzymes.

### 2.2. NAD^+^ Consumption

**Figure 2:**
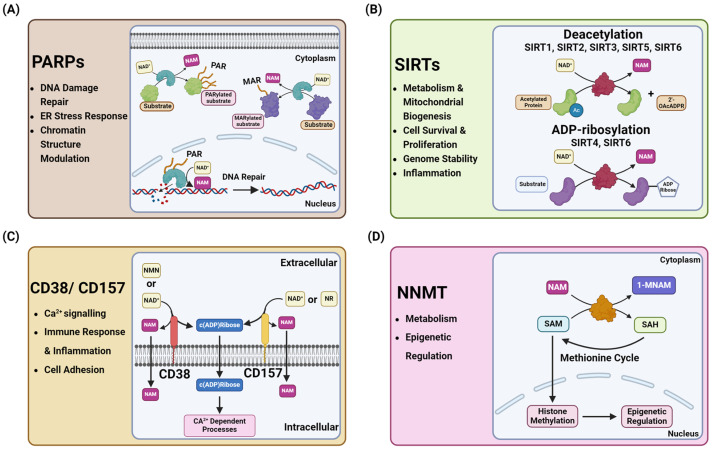
Reaction schemes for NAD^+^-consuming enzymes. A general reaction mechanism for the four types of NAD^+^-consuming enzymes. (**A**) Poly(ADP-ribosyl) polymerases (PARPs) are responsible for protein modification and DNA damage repair through their ability to form poly(ADP-ribose) (PAR) or mono(ADP-ribose) (MAR) chains. (**B**) Sirtuins (SIRTs) regulate various processes related to metabolism, DNA damage, inflammatory response, and general cell survival through the deacetylation of their substrates. (**C**) CD38 and CD157 are membrane-bound cADP-ribose synthases with glycohydrolase and ADP-ribosyl cyclase activities that regulate cell adhesion and Ca^2+^ signaling. (**D**) Nicotinamide N-methyltransferase (NNMT) utilizes the NAM produced in the salvage pathway to control histone methylation through the conversion of SAM to SAH, which simultaneously produces 1-MNAM. Created with BioRender.com accessed 15 August 2024.

NAD^+^ acts as a crucial cofactor in metabolic processes and is also required for several enzymatic reactions. Most notably, NAD^+^ is reduced to NADH or NAD^+^ to NADPH in several reactions during glycolysis and oxidative phosphorylation [31]. These reactions themselves are indicative of the pertinence of these redox couples in maintaining cellular metabolism and redox homeostasis. Yet, the NAD^+^ levels in cells are often regulated by its consumption through enzymatic cleavage to produce NAM, which is subsequently shuttled into the NAD^+^ salvage pathway to resynthesize NAD^+^ [32]. The predominant consumers can be categorized into four classes: poly (ADP-ribose) polymerases (PARPs), Sirtuins (SIRTs), CD38 and CD157 (two cADP-ribose synthases (cADPRs)), and nicotinamide N-methyltransferase (NNMT) (Figure 1C and Figure 2) [13]. These enzymes utilize NAD^+^ in order to maintain various cellular signaling pathways that include but are not limited to DNA repair, the inflammatory response, and post-translational modifications [20].

#### 2.2.1. Poly (ADP-Ribosyl) Polymerases (PARPs)

The human PARP enzyme family is composed of 17 unique proteins that are responsible for cleaving NAD^+^ to produce NAM and ADP-ribose [33]. The ADP-ribose is then used to build single or covalently linked polymers by the PARP enzymes, referred to as mono(ADP-ribosyl)ation or poly(ADP-ribosyl)ation, respectively [19]. Nuclear PARP enzymes are activated in response to single-stranded breaks in DNA, a process referred to as base excision repair (Figure 2A). PARP1, PARP2, and PARP3 are the members that are unique in their DNA-dependent activation, making them key regulators in DNA repair pathways [34,35]. The DNA-dependent PARP enzymes bind tightly to the DNA break and begin to build the poly(ADP-ribose) (PAR) chains to help build the scaffold that recruits DNA repair enzymes to repair the damaged DNA [36]. PARP1 in particular is a major consumer of NAD^+^, with it accounting for close to 90% of the total PARP activity [32]. However, as mentioned before, the other members of the PARP family are unique in their localizations, structure, and function [37].

Though PARP1 and PARP2 are known for their poly(ADP-ribosyl)ation (PARylation), most of the other PARP enzymes are monoenzymes responsible for catalyzing mono(ADP-ribosyl)ation (MARylation) reactions [38]. PARP7 (TiPARP) is one of the mono(ADP-ribosyl) transferases found in both the cytosol and nucleus. It was found to modify hundreds of different proteins, with targets identified through an orthogonal assay system using NAD^+^ analogs [39]. Of its known functions, PARP7 has been shown to modify α-tubulin on microtubules in the cytoskeleton of cancer cells [37] and to act as a negative regulator of type 1 interferon (IFN) signaling in tumor microenvironments [40]. Another member with a cytosolic localization is PARP16: a tail-anchored endoplasmic reticulum transmembrane protein functioning as an ER stress sensor [41]. PARP16 is pertinent in the activation of endoplasmic reticulum (ER) stress sensors such as PERK and IRE1α through ADP-ribosylation during the unfolded protein response (UPR) [41]. With PARPs being responsible for the regulation of various cellular stress responses as well as DNA damage repair pathways, the necessity of sustained NAD^+^ levels become pertinent for PARP function and ultimately cell survival in high-stress environments.

#### 2.2.2. Sirtuins (SIRTs)

Sirtuins are NAD^+^-dependent deacetylase enzymes that are also responsible for cleaving NAD^+^ to produce NAM and transfer acetyl groups from the lysine residues on a target protein onto an ADP-ribose molecule [42]. This family of enzymes in mammals is comprised of seven homologs (SIRT1-7) with different cellular localizations. These enzymes are ubiquitously active in cells, with SIRT1 and SIRT2 being responsible for nearly a third of the total NAD^+^ consumption in basal-condition cells [43]. SIRTs can deacetylate proteins including histones and various transcription factors and influence the signaling and metabolic pathways that maintain cellular homeostasis [19]. For example, SIRT1 is localized to the nucleus and has been heavily studied regarding its influence on DNA transcription, DNA damage repair, the cell cycle, and inflammatory pathways (Figure 2B) [44]. These enzymes can help to protect DNA from oxidative stress and damage; moreover, sirtuins have been directly linked to inhibiting senescence and allowing for increased cell division, which ultimately leads to tumorigenesis [45]. Their key role in ensuring cell survival has made SIRTs a subject of focus as a therapeutic target to prevent their effects on promoting cancer cell survival and proliferation [46].

#### 2.2.3. CD38 and CD157: cADP-Ribose Synthases (cADPRs)

CD38 and CD157 are cell-surface anchored, dual-receptor enzymes that are genetically homologous and have multifunctionality, allowing for both glycohydrolase and ADP-ribosyl cyclase activities [47]. This allows for CD38 and CD157 to utilize their glycohydrolase activity to cleave NAD^+^ into NAM and ADP-ribose and use their ADP-ribosyl cyclase activity to form cyclic ADP-ribose, a key messenger molecule in Ca^2+^ signaling (Figure 2C) [48]. Though they belong to the ADP-ribosyl cyclase family and share similar enzymatic function as well as their ability to be antigens [49], they vary in their structure, localization, and overall role. CD38 is a transmembrane protein that is universally expressed, particularly during inflammation [50], and an adhesion receptor that interacts with CD31 to mediate immune cell trafficking [51]. CD157 is a glycophosphatidylinositol-anchored protein that was originally identified as an Mo5 myeloid differentiation marker but has since been understood to have a much more diverse function in immunity that is not limited to just the myeloid compartment [52]. However, CD157’s possible cell surface receptor activity and its overall role in human immunity have yet to be fully understood [53].

#### 2.2.4. Nicotinamide N-Methyltransferase (NNMT)

As previously described, when NAD^+^ is consumed, the NAM that is produced is most often recycled through the salvage pathway (Figure 1C). However, another pathway exists branching from the salvage pathway. Here, NAM is methylated by nicotinamide N-methyltransferase (NNMT) to form 1-methyl-nicotinamide (1-MNAM), which can be further metabolized or excreted (Figure 1C) [54,55]. The reaction is dependent upon *S*-adenosyl-L-methionine (SAM) as a methyl doner to produce 1-MNAM and *S*-adenosyl-L-homocysteine (SAH) (Figure 2D) [56]. The 1-MNAM product is able to be oxidized by aldehyde oxidase (AOX) to produce two metabolites: *N*1-methyl-2-pyridone-5-carboxamide (2-pyridone) and *N*1-methyl-4-pyridone-3-carboxamide (4-pyridone) [57]. The 1-MNAM, 2-pyridone, and 4-pyridone are ultimately excreted in urine [56]. Due to its dependency on SAM as a cofactor, it is evident that NNMT plays a key role in maintaining the SAM:SAH ratio, often referred to as the “methylation index”, which is indicative of the hyper- or hypo-methylation of histones in cells [58]. This is indicative of NNMT’s greater role in regulating signaling pathways through a direct influence upon DNA transcription in cells. Evidence suggests that NNMT not only could regulate intracellular NAD^+^ or NAD^+^-dependent reactions but also play a greater role in several signaling pathways [59].

## 3. NAD^+^ Metabolism in Gynecologic Cancer

### 3.1. NAD^+^ Biosynthesis Pathway in Gynecologic Cancers

With their most distinctive feature being accelerated and unregulated growth, gynecologic cancer cells, like many other cancers, require altered metabolism to maintain this phenotype [60]. In cancer cells, the NAD^+^ levels are much higher compared to normal cells, which is likely due to an upregulation of NAD^+^ synthesis [5]. To meet the peak NAD^+^ requirements of both redox and non-redox processes, NAD^+^ is produced from dietary precursors such as tryptophan and forms of vitamin B3, as described previously (Figure 1) [14]. In this section, we describe how enzymatic activity in the NAD^+^ biosynthesis pathways shift during tumorigenesis and the influence they have over the tumor microenvironment and disease progression.

#### 3.1.1. Preiss–Handler Pathway: Nicotinic Acid Phosphoribosyl Transferase (NAPRT)

**Figure 3:**
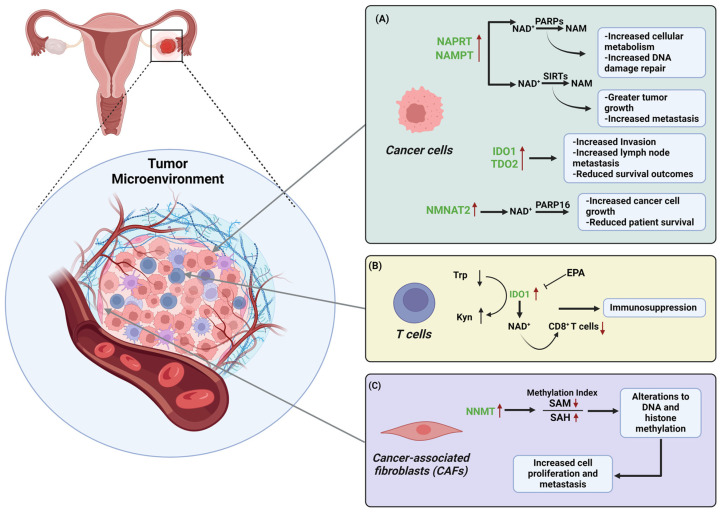
Summary reaction schemes depicting the influence NAD^+^ biosynthesis pathway reactions on the tumor microenvironment (TME). The TME often consists of (**A**) cancer cells, (**B**) T cells, and (**C**) cancer-associated fibroblasts. The interaction between NAD^+^-consuming enzymes and different cell types in the TME regulates various processes including tumorigenesis, progression, and metastasis in gynecologic cancers. Thus, the way NAD^+^ levels are maintained, in addition to the activity of the biosynthesis pathways, will trigger various processes in the context of the gynecological cancer tumor environments. Note. “↑” indicates “increase in expression” and “↓” indicates “decrease in expression levels”. Created with BioRender.com accessed 22 August 2024.

NAPRT has been shown to have heightened expression in several prevalent cancer types, including ovarian cancer [61,62]. Mutations in breast cancer gene 1 and 2 (BRCA1 and 2) increase the risk of developing ovarian cancer due to dysregulation of DNA damage repair. This is because BRCA genes play a role in in DNA double strand break repair, which is essential for the homologous recombination repair (HRR) pathway [63,64,65,66]. In epithelial ovarian cancer, it was found that high NAPRT expression was correlated with BRCA gene expression [67]. Consistent with these findings, NAPRT was found to have a role in DNA repair processes in the deficit of BRCA2 in response to chemotherapeutics [68]. It was then suggested that in cancers with defective HRR, including ovarian cancer, an increase in NAPRT expression confers a selective advantage by ensuring the maintenance of sufficient NAD^+^ levels, allowing for PARP activity and cellular protection from oxidative stress and DNA damage (Figure 3A) [68]. Due to NAPRT overexpression potentially causing resistance to DNA-damaging agents, the enzyme could act as a possible therapeutic target for patients with BRCA-mutated ovarian cancer to prevent or reverse treatment resistance.

#### 3.1.2. De Novo Pathway: Indoleamine-2,3-dioxygenase (IDO) and Tryptophan-2,3-dioxygenase (TDO)

Within the tumor microenvironment (TME), various types of immune cells have abnormal functions in response to the environment, thus inducing a loss of immune cell response and anti-tumor ability [69,70]. Tumor associated macrophages (TAMs), cancer-associated fibroblasts (CAFs), and regulatory T cells (Tregs) are examples of immunosuppressive cells that are responsible for the secretion of cytokines and other factors that help promote tumor immune escape in ovarian cancer, which presents challenges for effective immunotherapy [71,72,73]. IDOs and TDOs are intracellular enzymes that are responsible for the rate limiting step of the de novo biosynthesis pathway (Figure 1A); however, they are also capable of mediating various tumor-induced immunosuppressive mechanisms [74]. It is currently postulated that IDO1, IDO2, and TDO activity can cause near total depletion of cellular tryptophan, which in turn triggers an accumulation of uncharged tRNAs and consequently activates the GCN5 kinase pathway and causes T cell dysfunction [75]. However, only a few studies have been able to show the role of both IDOs and TDO in immunosuppression within human gynecologic cancers.

Okamoto et al. demonstrated that high expression of IDO in serous types of ovarian cancer was associated with poorer survival outcomes in patients that received paclitaxel-based chemotherapy [76]. When investigating the role of IDO in ovarian cancer, it was shown that IDO-overexpressing tumor cells potentiate immunosuppressive activity [77]. In a study conducted by Inaba et al. that utilized an in vivo xenograft ovarian cancer model, it was demonstrated that tumor progression was enhanced upon elevated expression of IDO because of its inhibitory effect on host immune cells. The authors were also able to show that high-IDO-expressing tumors showed significantly lower CD8+ tumor-infiltrating lymphocyte (TIL) numbers compared to lower or non-expressing IDO patient samples [77]. In endometrial cancer, similar results were observed, with high IDO expression in tumor cells correlating with greater tumor cell invasion and increased lymph node metastasis and reducing both overall survival and disease-free progression (Figure 3B and Table 1) [78]. In the context of targeting immunosuppression in patients, IDO inhibition has been shown to yield limited therapeutic efficacy in cancer-patient clinical trials when administered as a monotherapy [79]. A recent study by Odunsi et al. also found that the metabolic adaptations triggered by IDO1 blockade increased the level of NAD^+^ in the TME, which in turn limited T cell function. Additionally, the authors showed that the combination of the IDO1 inhibitor epacadostat and a purinergic receptor antagonist was able to rescue T cell function and proliferation in mouse models for ovarian cancer [80].

Like IDO1, TDO2 is expressed at higher levels in cancer tissue than in normal tissue [94]. Notably, TDO2 has been reported to be expressed in different cancer cells, including breast, ovarian, glioma, and hepatic carcinoma [74,95,96]. When comparing the expression of TDO2 in ovarian cancer tissue to that of normal fallopian tube tissue, Zhao et al. found that the mRNA levels were significantly higher. The authors also noted that overexpressing TDO2 in cancer cell lines was associated with increases in migration and invasion; TDO2-specific siRNA knockdown had the opposite effect [97]. Additionally, a study by Smith et al. showed that the RNA expression, when compared between ovarian cancer patients from the Tothill cohort (n = 293) [81], was significantly associated with disease stage, recurrence, and survival [98]. Thus, the dual inhibition of IDO/TDO could be regarded as a unique approach for the targeting the de novo pathway.

#### 3.1.3. Salvage Pathway: Nicotinamide Phosphoribosyl Transferase (NAMPT)

As the rate-limiting enzyme in the NAD^+^ biosynthetic salvage pathway, NAMPT influences intracellular NAD^+^ levels through its pivotal role in NAD^+^ biosynthesis from nicotinamide [10,99]. In the context of cancer biology, NAMPT is often regarded as a potent oncogene (Figure 3A and Table 1) [100]. Several studies have highlighted the upregulated expression of NAMPT in various cancers, including colorectal, breast, ovarian, endometrial, prostate, and pancreatic cancers, with higher NAMPT expression often being correlated with poorer survival outcomes [100,101,102,103,104]. In ovarian cancer, increased NAMPT expression has been found to be linked to tumor lymph node metastases, invasion, advanced clinical stage, and poor survival outcomes [101].

BRCA1 knockdown was found to effectively increase NAMPT levels in ovarian cancer cells and primary non-BRCA1-mutated cells, suggesting a critical role for NAMPT in BRCA1-related NAD^+^ synthesis in ovarian cancer [105]. Numerous studies have shown that changes in NAMPT expression occur with the development of drug resistance to targeted therapies [100,106,107,108]. Platinum resistance is a lasting issue for most ovarian cancer patients, with most platinum-based therapies operating by inducing cellular senescence and causing the induction of cancer-stem-like cells as a response [109]. This new stem-like phenotype ultimately causes chemoresistance, as ovarian cancer cells will adapt a quiescent phenotype allowing for an autophagic state [110]. When analyzing the NAMPT derived from patient ascites, the authors found that the NAMPT was inducing an increased migratory ability in ovarian cancer cells. This phenotype was observed to be caused by the activation of Rho-ROCK-mediated signaling that induced actin polymerization [111]. A study by Kudo et al. has provided evidence of a link between using KPT-9274—a dual NAMPT and PAK4 inhibitor—and the reduction of inflammation, decrease in cell growth, and downregulation of DNA-repair-related genes in platinum-resistant 3D-cultured ovarian cancers spheroids [112]. Another study by Narcelli et al. showed that treatment using the NAMPT inhibitor FK866 in combination with cisplatin treatment reduces tumor growth, delays tumor relapse, and improves the survival outcomes of cisplatin-treated epithelial ovarian cancer (EOC) in pre-clinical models [109].

In endometrial cancer, NAMPT expression is shown to be relatively higher in endometrial carcinoma patient tissues when compared to normal tissues and that high NAMPT levels lead to poor survival outcomes [113]. Additionally, eNAMPT levels were found to be elevated in the serum from endometrial cancer patients, and this was found to be correlated with NAMPT expression in the tumor tissue [113]. In pre-clinical models, NAMPT enhanced endometrial cancer progression by targeting the PI3K/AKT and MAPK/ERK signaling pathways, altering its progression through the G1/S phase of the cell cycle (Table 1) [114]. In vivo xenograft models demonstrated that NAMPT-treated mice showed enhanced tumor growth in endometrial carcinoma, exhibiting a high Ki-67 proliferation index [114].

Though NAMPT is greatly responsible for the conversion of NAM to NMN, it is not the only source of NMN that cells have access to. Cells can uptake NMN directly from the extracellular environment, as well as use extracellular NR as previously mentioned. Ecto-5′-nucleotidase CD73 is a GPI-anchored glycoprotein on the extracellular side of the plasma membrane [115,116]. CD73 consumes NMN and converts it to NR [117], which then becomes internalized and converted back to NMN by nicotinamide riboside kinases (NRK1/2) (Figure 1C) [118]. The CD73 found in tumor immune environments is overexpressed in different cancers, including ovarian cancer [119]. In cancer cells, CD73 generates adenosine, an immunosuppressive metabolite, thereby affecting the anti-tumor T-cell response [120]. CD73 activity leads to an enhancement in DNA damage repair and causes chemotherapy resistance through its NR production [121]. High CD73 expression has been reported to be associated with worse clinical outcomes in ovarian cancer patients and may lead to the development of metastasis [119]. In fibroblasts, increased expression of CD73 enhances tumor immunosuppression and promotes tumor growth [119]. CD73-mediated adenosine production can also promote tumor cell migration and metastasis by activating adenosine’s A2A and A2B receptors [82]. The inhibition of the adenosine A2A and A2B receptors (A2AR/A2BR) was found to enhance the immune system’s ability to exert an anti-tumor response against cancer cells; conversely, stimulating the A2A and A2B receptors limits T-cell proliferation and cytotoxicity [120,122,123].

#### 3.1.4. Salvage Pathway: Nicotinamide Mononucleotide Adenylyl Transferases (NMNATs)

In ovarian cancer cells, ectopic NMNAT1 expression was seen to reduce the cytosolic NAD^+^ levels but enhance the nuclear NAD^+^ levels [38]. The elevated levels of NMNAT2 in ovarian cancer cells have been shown to decrease the accumulation of toxic proteins through the NMNAT2-NAD^+^-PARP16 pathway, which then supports cancer cell proliferation (Figure 3A) [38]. Depletion of NMNAT2 results in an increase in the polysome loading of mRNAs containing a specific stem-loop element in their 3′ UTRs. This increase enhances protein synthesis, causing an increase in the accumulation of toxic protein aggregates, which then inhibits ovarian cancer cell growth (Table 1) [38]. Ovarian cancer cells use the NMNAT2-PARP16 interaction to balance proteostasis while accelerating the cell growth that often relies on increased ribosome biogenesis and protein synthesis [41]. Moreover, high NMNAT2 expression is associated with higher tumor grades and is linked to poorer progression-free survival in ovarian cancers [38]. This dependence on NMNAT2 creates a vulnerability that can be targeted to inhibit cancer cell growth.

### 3.2. NAD^+^ Consumers in Gynecologic Cancers

With NAD^+^ levels being so closely linked to tumorigenesis and tumor progression, a better understanding of the changes to NAD^+^ consumers in gynecologic cancers can give us insight into how cancer cells differentiate from their parent tissue cell type. In the following section, we will discuss the changes in NAD^+^ consumers in gynecologic cancers and note their distinct roles in gynecologic cancer progression (Table 1).

#### 3.2.1. PARPs in Gynecologic Cancers

PARP1, the most studied member of the PARP family, plays a crucial role in repairing single-strand breaks (SSBs) and is activated in response to DNA damage, thus ensuring the safeguarding of DNA integrity and maintaining genomic stability [124]. When activated in cells experiencing DNA damage or external stress, PARP1 consumes substantial amounts of NAD^+^ during the DNA repair process, leading to NAD^+^ depletion [125]. PARP1 induces apoptosis by activating p53 under normal conditions in response to severe DNA damage [84]. However, excessive activation of PARP1 following genotoxic exposure can deplete NAD^+^ and ATP levels, disrupting sirtuin activity and cellular homeostasis and ultimately leading to cell death [85,126]. PARP1 can function as either a tumor promoter or suppressor depending on its degree of activation [127,128]. Still, many tumors upregulate PARP1 to aid in DNA damage repair, particularly under the influence of cellular stressors like radiotherapy and chemotherapy (Table 1). Elevated PARP1 expression is commonly observed in various carcinomas such as breast, ovarian, and lung cancers and non-Hodgkin’s lymphoma [84]. When examining patient epithelial ovarian cancer tissue, it was found that 73.3% of a 60 patient cohort had overexpression of PARP1 when measured using immunohistochemistry [87]. Though the mechanism has yet to be explored, studies have shown that platinum-treatment-resistant epithelial ovarian cancer (EOC) tissue has even greater levels of PARP1 expression, leading to speculation of its potential as a marker for predicting poor prognosis and platinum resistance [87,129].

In addition to PARP1, other members of the PARP family play distinct roles in ovarian cancers. The cancer cells’ accelerated growth creates the necessity for the proper regulation of protein synthesis and other metabolic processes to ensure cell survival. Previous studies have also shown that to achieve proper cell growth and survival, many transformed cancer cells have altered levels of proteotoxic stress [86]. Many ovarian cancer cell lines have shown overexpression of NMNAT2, indicating an increase in cytosolic NAD^+^ levels as well as cell growth [38]. Due to the direct endoplasmic reticulum localization of PARP16, the direct interaction between PARP16 and NMNAT2 influences protein synthesis levels. When investigating the compartmentalization of NAD^+^ synthesis in ovarian cancer cells, Challa et al. identified a pathway where NMNAT2 and PARP16 together regulate ribosome function and protein homeostasis in ovarian cancer cell lines (Figure 3A) [38]. As mentioned in the previous section, the activity of PARP16 is directly influenced by the overexpression of NMNAT2, which increases the MARylation of ribosomal proteins by PARP16, leading to the inhibition of protein synthesis and preventing the protein aggregation that would lead to greater cellular stress that reduces growth and increases cell death [38]. Similar to PARP16, MARylation of key cytosolic proteins by PARP7 has been found to regulate the biology of ovarian cancer cells. The expression levels of PARP7 differs between normal and tumor tissues, with malignant ovarian cancer cells showing higher expression of PARP7 [130,131]. PARP7 was found to MARylate α-tubulin, causing the destabilization of microtubules in the cytoskeleton. This has led to the hypothesis that PARP7 is linked to greater motility and migration in ovarian cancer cells [37].

When NAD^+^ homeostasis is dysregulated, there is a direct effect on PARP activity and DNA damage repair. PARPs have been targeted using inhibitors such as olaparib, niraparib, rucaparib, and veliparib to prevent the activation of PARPs in diseases like cancer where the DNA damage is high in cells [132]. The use of PARP inhibitors (PARPis), particularly PARP1 inhibitors, has been shown to suppress cell viability and motility in EOC cells and could potentially increase sensitivity to platinum treatments and other chemotherapeutics [133]. Current studies are also focused on developing inhibitors for the PARP monoenzymes that are key therapeutic targets for ovarian cancers such as PARP7 and PARP16 [134,135]. These topics will be discussed in further detail in the coming sections.

#### 3.2.2. SIRTs in Gynecologic Cancers

SIRT1 has been shown to be upregulated in multiple tumor types, including gynecologic cancers, suggesting a strong association of its expression to tumorigenesis [45,46,88,91]. Across gynecologic cancers, SIRT1 expression was shown to have high heterogeneity across histological subtypes, making characterizing its role difficult [136]. However, several studies have suggested that SIRT1 plays a greater role in tumorigenesis than in cancer progression [89,137]. In endometrial cancer, two subtypes exist, with type 1 making up for about 80% of cases and having more favorable survival outcomes and type 2 being rarer but considered more aggressive [138]. In a 2016 study involving 76 EC patients of multiple types, it was shown that SIRT1 mRNA is significantly reduced, yet the SIRT1 protein is overexpressed; however, a correlation between SIRT1 levels and EC type, grade, and stage could not be established [90]. In cervical cancers, SIRT1 begins to be overexpressed in squamous intraepithelial neoplasia and squamous cell carcinoma and continues to increase as the disease progresses [91]. With nearly all cases of cervical cancer having been caused by high-risk HPV, understanding the interaction between HPV infection and tumorigenesis has illuminated the role of SIRT1 in aiding and protecting HPV-infected cells and promoting tumorigenesis (Table 1). It was shown that in the initial stages of viral infection, SIRT1 acts as part of a protein complex with the HPV proteins E1 and E2 to ensure proper incorporation and replication of viral DNA [139]. The viral proteins E6 and E7 play a significant role, with E6 triggering lower expression of p53 and E7 altering the expression of retinoblastoma protein (pRb), allowing for an increase in unmediated cell growth [92]. Specifically, HPV E7 is capable of upregulating SIRT1, inducing cell growth; moreover, knocking out SIRT1 or E7 was shown to trigger apoptosis, indicating the possible anti-apoptotic role of SIRT1 as well as the heavy dependency on E7 for cell survival [92].

#### 3.2.3. CD38 and CD157 in Gynecologic Cancers

Zhu et al. conducted a recent study where the authors analyzed CD38 mRNA expression levels in normal ovaries and epithelial ovarian cancer (EOC) using online datasets like GEPIA, Oncomine, and TISIDB. It was found that CD38 expression in EOC is significantly higher in borderline ovarian tumors when compared to normal tissue [140]. Variations in CD38 expression across the immune subtypes of ovarian cancer were noted, with the C2 subtype (IFN-γ dominant) showing the highest levels [140]. This analysis emphasizes CD38’s strong association with immunological characteristics in the tumor microenvironment. Furthermore, high CD38 expression is correlated with better survival outcomes in EOC, particularly in stages III and IV and grades II and III [140].

Studies have shown that CD157 expression in ovarian cancer cells enhances tissue invasion and cell migration [141], thus playing a pivotal role in promoting ovarian cancer metastasis by regulating the interaction of ovarian cancer cells with the mesothelium and extracellular matrix proteins through its expression [142]. CD157-overexpressing ovarian cancer cells also exhibit mesenchymal traits, promoting tumor cell proliferation while reducing apoptosis [141]. Furthermore, high CD157 expression was seen to be correlated with increased malignancy and a higher risk of relapse in ovarian cancer [143]. When comparing serous ovarian cancer primary tissue cells to those obtained from the patient’s ascites, Ortolan et al. were able to note that nearly all the primary tumor cells expressed CD157, yet only 10% of the cells isolated from ascites expressed CD157. Furthermore, the authors found that cells were able to re-express CD157 after being cultured on plates, allowing for the theory that the expression is adhesions dependent. Thus, regarding the prospect of reversing the trend that was seen, the authors proposed targeting CD157 with monoclonal antibodies in order to reduce tumor cell adhesion to extracellular proteins like collagen, fibronectin, and laminin [143].

#### 3.2.4. NNMT in Gynecologic Cancers

NNMT has been found to be upregulated in multiple cancer cell types, causing a decrease in the SAM:SAH ratio through consumption of SAM, ultimately leading to an altered epigenetic state [144]. Its role in directly regulating the SAM:SAH ratio in cells implicates NNMT in playing a significant role in controlling the gene expression within ovarian cancer cells and cancer-associated fibroblasts (CAFs) (Figure 3C). Through proteomic analysis, NNMT has been identified as a key regulator in ovarian cancer proliferation and metastasis, as well as changes in tumor stroma gene expression within CAFs [93]. The expression of NNMT is shown to be increased in peritoneal stroma and omental metastases compared to benign fallopian tube ovarian stroma [93]. Because NNMT can act as a methyl sink and predominant SAM consumer, the upregulation of this enzyme in ovarian cancer cells and CAFs shows the potential for the metabolic and genomic reprogramming that is associated with tumor metastasis and aggression (Table 1). The upregulation of NNMT then alters metabolism and sensitizes ovarian cancer cells to mitochondrial metabolic targeting agents without affecting proliferation [145].

Additionally, BRCA1 has been found to regulate NNMT expression by occupying its promoter. The depletion of BRCA1 leads to mitochondrial respiration defects and reduced ATP levels due to a lower mitochondrial DNA copy number. The decreased ATP levels from BRCA1 depletion are mainly due to NNMT upregulation, making NNMT a potential therapeutic target in BRCA1-deficient ovarian tumors [145,146]. It has also been shown that NNMT directly upregulates connective tissue growth factor (CTGF) expression by reducing the methylation of the CTGF promoter, revealing a novel pathway linking metabolism and mesenchymal gene expression. This pathway highlights the mechanisms underlying cancer cell survival and metastasis under nutrient-deficient conditions [147]. In both glucose-dependent and -independent ovarian cancer cells, ectopic ZEB1 enhances NNMT expression and the expression of EMT-associated genes such as MMP2, CTGF, and SPARC. The ZEB1-NNMT axis promotes EMT, ultimately allowing for phenotypic and metabolic plasticity [148]. High NNMT expression in glucose-restricted ovarian cancer cells enables metabolic adaptations by utilizing alternative sugars and methylated substrates [144]. Accumulating evidence has shown that a knockdown of NNMT reduces both tumorigenesis and chemoresistance and further research is needed to explore NNMT’s potential as a therapeutic target for improving anti-cancer treatments.

## 4. Targeting NAD^+^ Metabolism in Gynecologic Cancers

The upregulation of NAD^+^ synthesis has been found to be associated with cancer development and progression [149]. Inhibiting NAD^+^ synthesis and targeting NAD^+^ consumers show promise in cancer treatment, likely due to the differing roles of these enzymes [150]. NAD^+^ synthesis enzymes mainly influence tumor cell behaviors, while NAD^+^-consuming enzymes affect the immunosuppressive tumor microenvironment [149]. Enzymes such as Sirtuins, PARPs, CD38, and other consumers influence critical cellular functions and are closely linked to cancer progression, as described in the previous section [151]. Recent studies have revealed these enzymes’ roles in regulating cell metabolism, cytokine release, neoantigen expression, and modifying the tumor immune microenvironment, thereby exhibiting anticancer effects [83,152]. Drug development in targeting these enzymes is ongoing. Here, we summarize the recent advancements in inhibitors targeting the NAD^+^ biosynthesis and consumption pathways in gynecologic cancers (Table 2).

### 4.1. Targeting NAD^+^ Biosynthesis via the Salvaage Pathway—Targeting NAMPT

Nicotinamide phosphoribosyl transferase (NAMPT) regulates the intracellular NAD^+^ levels crucial for cellular redox reactions and NAD^+^-dependent enzyme function, and it also exhibits cytokine-like activity [19]. NAMPT influences metabolic disorders and cancer by modulating oxidative stress, apoptosis, lipid metabolism, inflammation, and insulin resistance [10]. NAMPT is overexpressed in various cancers due to the need to meet increased energy demands, driving cellular metabolism and responses to NAD^+^ depletion [150]. This overexpression, coupled with increased NAD^+^ levels, enhances cancer cell survival by making cells resistant to anti-cancer treatments [171]. Additionally, NAMPT-specific inhibitors lower NAD^+^ levels and the activity of metabolic pathways including glycolysis, the citric acid cycle, and OXPHOS, thereby helping to suppress cancer cell proliferation [172]. Several reports have also indicated a link between elevated NAMPT levels and negative clinical outcomes including reduced survival rates across various types of cancer [173,174]. Specifically, an analysis using TCGA datasets by Kudo et al. found that an elevated level in NAMPT expression was consistent with worse overall survival in patients with ovarian, endometrial, and cervical cancers [112]. As a result, NAMPT has been a target for the development of a number of inhibitors over the past few decades. NAMPT inhibitors have demonstrated significant anticancer activity in both in vitro and in vivo cancer models [172]. Targeting NAMPT for oncology has led to the development of compounds with improved inhibitory effects, such as FK866 (also known as (E)-Daporinad), CHS828, GEN617, OT-82, GMX1777, and other cytotoxic agents [175,176,177]. Among the identified NAMPT inhibitors, the prototypical compound FK866 and CHS-828, with its prodrug GMX1777, have been evaluated in early-phase clinical trials involving cancer patients. Ongoing research aims to develop NAMPT inhibitors with enhanced therapeutic efficacy and optimized dosing strategies [178]. In this section, we will highlight the antitumor effects of NAMPT inhibitors both in vitro and in vivo in gynecologic cancer models, as well as advancements in their clinical applications.

#### 4.1.1. FK866

(E)-N-[4-(1-Benzoylpiperidin-4-yl)butyl]-3-(pyridin-3-yl)acrylamide (FK866) was the first NAMPT inhibitor reported by Hasmann et al. in 2003 [175]. FK866 has an IC50 of 1nM and binds to the same binding pocket as nicotinamide but with higher affinity, acting as a partially noncompetitive inhibitor to NAMPT. FK866 indirectly suppresses mitochondrial respiratory activity while selectively inhibiting NAMPT, thereby causing a gradual depletion of NAD^+^ [175]. This conclusion was further supported by Gogola-Mruk et al. in ovarian cancer spheroids, where the authors were able to show that visfatin, another name for NAMPT, increased ATP levels and mitochondrial activity, while its inhibition with FK866 reversed these effects [153].

##### FK866 and PARP Inhibitors

Poly(ADP-ribose) polymerase inhibitors (PARPis) are the standards of care for the treatment of advanced and recurrent epithelial ovarian cancer [179]. PARPis target the PARP1 protein that synthesizes chains of poly(ADP-ribose) (PAR) onto its target DNA [180]. PARP1 cleaves NAD^+^ into nicotinamide (NAM) and ADP-ribose (ADPr), attaching the ADPR moieties to its target DNA damage. NAM is recycled to form NAD^+^ through the salvage pathway via NAMPT and the NMNATs [181]. This process is critical for DNA damage repair, chromatin remodeling, and cell death [180]. Due to the long exposure to this drug treatment, resistance to PARPis has been observed as leading to recurrence; efforts have been made to study the mechanisms and ways to overcome resistance to PARPis [182]. FK866 was found to sensitize PARPi-resistant cell-based models to the treatment of PARPis [155]. In this study, olaparib-resistant cell lines were created by exposing ovarian cancer cell lines to increasing concentrations of olaparib. Compared to single drug treatments of olaparib or FK866, the combined regiment resulted in at least twice the level of cleaved caspase-3 expression and about five times the level of γ-H2AX, markers for apoptosis and DNA damage, respectively. Synergy was observed in patient-derived high grade serous ovarian cancer organoid models when the combined regimen was used. In the mouse xenograft models, at the end of the treatment period, the relative tumor volume of the mice treated with both drugs was slightly more than half of those treated with either drug alone [155], suggesting that the combination of an NAMPTi and PARPi could provide a new strategy for the treatment of ovarian cancer, especially in PARPi-resistant settings.

##### FK866 and CD73 Inhibitor

CD73 converts NAD^+^/NMN to nicotinamide riboside (NR), which then crosses the plasma membrane and is phosphorylated to form NMN through the intracellular biosynthesis of NAD^+^ (Figure 1C) [82]. CD73 expression is increased in several types of cancer [183] and is associated with poor prognosis in patients with high grade serous ovarian cancer [119], leading Sociali et al. to investigate the effect of blocking both NAMPT and CD73 in an ovarian cancer mouse model [156]. CD73 was found to produce NR from extracellular NMN, which counteracted with the antitumor effect of NAMPT inhibition by FK866. When mice were treated with adenosine 5′-(α,β-methylene)diphosphate (APCP)—a CD73 inhibitor—and FK866, the level of NAD^+^, NMN, and ATP in mouse tumors statistically decreased two to threefold when compared to either treatment alone. Necrotic areas in the mice treated with the combined regiment were also three to five times higher than in mice treated with either inhibitor alone; additionally, these mice had a small advantage in overall survival. This approach significantly enhanced the anticancer efficacy of FK866 [156].

##### FK866 and Chemotherapy

In gynecologic cancers, FK866 treatment was shown by Nacarelli et al. to result in better survival outcomes in mice transplanted with the ovarian cancer cell line OVCAR3 when combined with cisplatin [109]. In this study, the authors demonstrated that inhibition of NAMPT resulted in the suppression of senescence-associated cancer-stem-like cells (CSCs), which are induced by platinum-based chemotherapy and can contribute to chemoresistance in ovarian cancer. The combination of FK866 and cisplatin also resulted in reduced tumor outgrowth and improved overall survival in mice bearing epithelial ovarian cancer [109].

#### 4.1.2. CHS828 and GMX1777

CHS828 is a cyanoguanidine that was first reported by Schou et al. in 1997 for its antitumor effects and has exhibited potent anticancer effects in lung and breast cancer cell lines [184]. The inhibitor was being utilized in clinical trials before FK866, but it was not until 2008 that Olesen et al. established its mechanism of action as an NAMPT inhibitor [185]. CHS828 acts as a competitive inhibitor of NAMPT, decreasing the cellular level of NAD^+^ and resulting in cell death and tumor regression [177]. Crystallographic analysis revealed that, similar to FK866, CHS828 binds to the NAMPT tunnel cavity [186]. Nacarelli et al. showed that CHS828 also demonstrated antitumor activity in OVCAR3 cells and organoid models using human ovarian cancer tissues when combined with cisplatin by suppressing senescence-associated cancer-stem-like cells [109]. To address the solubility and pharmacokinetic challenges observed in early clinical trials of CHS828, Binderup et al. developed several prodrugs during this period with improved properties [187]. Of these prodrugs, GMX1777 stood out as the most promising compound; it featured a tetraethylene glycol segment linked to the parent drug via a carbonate bond. This modification enhanced its solubility, facilitating rapid release upon intravenous administration in vivo [187].

Several early phase clinical trials including GMX 1777, CHS828, and FK866 have produced rather disappointing results; however, only two included gynecologic cancer patients. In 2002, Hovstadius et al. recruited 16 patients with solid tumors in a phase I study with CHS828, eight of whom had GYN cancers (leiomyosarcoma and ovarian carcinoma) [157]. The second trial with reported results that included GYN cancer patients was from von Heideman et al., where two out of a total of eight patients recruited had ovarian cancer [158]. Both trials showed relatively similar toxicity profiles, with thrombocytopenia being the most common dose-limiting toxicity. GMX1777 was used in a trial by Pishvaian et al. in patients with solid tumors [159]. Eight out of 19 patients recruited were female; however, it is unclear whether GYN disease sites were included. Thrombocytopenia, gastrointestinal hemorrhage, and skin rash were noted at higher doses. None of these early trials resulted in a significant clinical response, and the toxicity profile was rather significant. Interpretations of the phase I clinical trials are inherently complicated, as researchers had to use different strategies to adjust the dose and route of administration [158]. Additionally, the patients included in these trials had disease that had progressed through multiple lines of therapy, with poorer performance status to tolerate the drug itself [158]. In a review by Tang et al., the authors suggested that since the role of NAMPT was broad, it would be difficult to identify the patient population for the inhibitors, highlighting the need to identify better biomarkers for a targeted therapeutic strategy [188].

#### 4.1.3. KPT-9274

P21 activated kinase 4 (PAK4) plays a critical role in various signaling pathways in cancer, including the Wnt/β-catenin, RAS-ERK, and androgen/estrogen receptor pathways [189]. PAK4 is overexpressed across different cancer types and has been proposed as a biomarker for cancer [10,190]. KPT-9274 is a dual inhibitor, targeting both NAMPT and PAK4 [112]. Using 3D spheroids with ovarian cancer cells resistant to platinum-based therapy that mimic the CSC-enriched tumor masses floating intraperitoneally, Kudo et al. demonstrated that KPT-9274 inhibited NAD^+^ production via direct NAMPT inhibition and also reduced PAK4 activity, resulting in an NAD^+^-dependent decrease in the phosphorylation of S6 ribosomal protein, AKT, and β-Catenin [112]. In clinical trials, KPT-9274 was used in the PANAMA trial (NCT02702492), where patients with advanced solid tumors were recruited for the study [160]. This study was terminated in 2021, and the results have not been reported; it is also unclear whether any of the participants had GYN cancer. Despite promising preclinical results, NAMPT inhibitors have shown limited tumor responses in phase I/II clinical trials, suggesting the need for biomarkers to identify patients who may benefit from these inhibitors.

### 4.2. Targeting NAD^+^ Biosyntehsis via the Preiss–Handler Pathway—Targeting NAPRT

Besides NAMPT, enzymes in other NAD^+^ biosynthesis pathways have been considered as potential targets in gynecologic cancer cell lines. Nicotinic acid phosphoribosyl transferase (NAPRT) helps transfer a phosphoribosyl moiety from phosphoribosyl pyrophosphate (PRPP) to nicotinic acid (NA), generating nicotinic acid mononucleotide (NAMN) as part of the Preiss–Handler pathway (Figure 1B) [172]. The NAPRT gene is overexpressed in a subset of cancer types, including ovarian cancer [68]. Cancers dependent on the NAD^+^ salvage pathway and deficient in NAPRT are sensitive to NAMPT inhibitors, while cancers dependent on the Preiss–Handler pathway with high NAPRT expression are resistant. However, tumors relying on NAPRT for NAD^+^ synthesis can become sensitive to NAMPT inhibitors after NAPRT downregulation [68,149], highlighting the need for the development of NAPRT inhibitors. A study by Piacente et al. showed that 2-hydroxynicotinic acid (2-HNA), an NAPRT inhibitor, can sensitize ovarian cancer cells to treatment with an NAMPT inhibitor, resulting in the marked cell death of OVCAR5 cells in vitro, as well as OVCAR5 in xenograft-bearing mice [68]. Specifically, the levels of NAD^+^ expression when OVCAR5 cells were treated with FK866 and 2-HNA were about half those when treating with FK866 alone. Similarly, treating the cells with the combined regimen resulted in about ten times more cell death than treating the cells with FK866 alone [68]. Mice inoculated with OVCAR5 cells also survived approximately ten days longer when treated with FK866 and 2-HNA rather than with FK866 alone [68]. Recently, a new small-molecule NAPRT inhibitor (NAPRTi) identified through in silico methods has shown synergistic effects when combined with NAPRTi and FK866, suggesting its therapeutic promise. However, additional studies are necessary to validate their efficacy [191,192].

### 4.3. Targeting NAD^+^ Consumers

#### 4.3.1. PARP Inhibitors

As a major NAD^+^ consumer, PARP inhibitors are probably the most clinically utilized inhibitors of NAD^+^ metabolic pathways in treating GYN cancers. Olaparib was the first PARP inhibitor approved in 2014 following Study 42, which demonstrated its effectiveness as a monotherapy in patients with germline BRCA1/2 mutations who had had previously multiple lines of chemotherapy [161]. Olaparib was then brought to the front line as maintenance therapy in patients with BRCA-mutated ovarian cancer after SOLO1, a phase III clinical trials in 2018 [162]. Rucaparib was the second PARP inhibitor approved, receiving FDA accelerated approval in 2016 based on the findings of the ARIEL2 trial for patients with BRCA-mutated, relapsed ovarian cancer and, subsequently, approval as a maintenance therapy for patients with platinum-sensitive ovarian cancer based on the results of the ARIEL3 trial in 2018 [163]. Niraparib was the most recent PARPi to receive approval as a treatment for ovarian cancer, with the first approval for maintenance therapy happening in 2017 in the NOVA trial and its approval for first-line maintenance therapy in newly diagnosed ovarian cancer in the PRIMA trial [164]. PARP inhibitors are, overall, quite well tolerated, with common side effects including fatigue, anemia, and nausea. In 2022, the FDA approval was withdrawn for olaparib, niraparib, and rucaparib for patients with multiple-line therapies based on the findings from the ARIEL4, SOLO3, and QUADRA trials [165,166,167,168]. However, PARP inhibitors remain an important frontline maintenance therapy for patients with BRCA-mutated, platinum-sensitive ovarian cancer

Resistance to PARP inhibitors, both endogenous and acquired, has been identified to contribute to disease progression. A review by Guidice et al. discussed HR function restoration, DNA replication fork stabilization, BRCA reversion mutations, dissociation of PARP1 and PARG, and epigenetic modifications as some of the well-studied mechanisms of PARP inhibitor resistance [193]. Current studies are working to identify strategies to overcome resistance, including various combinations of PARP inhibitors and other pathway inhibitors [193].

#### 4.3.2. Sirtuin Inhibitors

A recent study explored the role of the novel SIRT1 inhibitor MHY2245 in regulating apoptosis and autophagic cell death in ovarian cancer cells, along with its underlying mechanisms [169]. This study was the first to assess the anticancer effects of MHY2245 on human ovarian cancer cells. Treatment with MHY2245 significantly reduced colony formation in SKOV3 cells compared to the controls, suggesting that SIRT1 enhances tumor formation and promotes expression of cell-cycle-related genes (Figure 2A). The mechanism through which MHY2245 exerts its cytotoxicity is by inhibiting SIRT1, thereby arresting the cell cycle at G2/M phase and inducing apoptosis [169]. Additionally, when inhibiting SIRT1 with MHY2245, a decrease in phosphorylated Akt was noted, suggesting that MHY2245 was involved in the downregulation of the PI3K/Akt/mTOR pathway [169]. The antitumor effects of MHY2245 were also demonstrated in an in vivo xenograft model, where MHY2245 significantly reduced the growth of SKOV3 cell tumors by about 30–40% [169]. This study suggests that MHY2245 has a potent activity against SIRT1 and holds promise for ovarian cancer therapy.

#### 4.3.3. NNMT Inhibitors

NNMT has been found to have elevated expression in peritoneal stroma and omental metastases in ovarian cancer, and its expression correlates with the aggressive behavior of ovarian cancers with poor prognosis [93,194]. NNMT is a potential target for cancer therapy development; there are currently three main types of NNMT inhibitors: competitive, bi-substrate, and covalent [195]. Among these existing inhibitors, 5-amino-1-methylquinolinium (5-amino-1MQ) was recently used in a study by Eckert et al. to demonstrate the critical role of NNMT in the tumor microenvironment in facilitating ovarian cancer growth and metastasis [93]. When CAFs were treated with 5-amino-1MQ, histone methylation increased, supporting the authors’ hypothesis that ovarian cancer progression via NNMT was driven by epigenetic changes in the stromal cells. Mice treated with 5-amino-1MQ after being injected with ovarian cancer HeyA8 cells were found to have decreased tumor burden by about threefold and increased stromal H3K27 methylation [93]. 5-Amino-1MQ was also used in another study on ovarian cancer cells by Huang et al. [170]. In this study, the authors found that expression of fat mass and obesity associated protein (FTO) increased the sensitivity of ovarian cancer cells to platinum both in vitro and in vivo. NNMT was found to be targeted by FTO, which was upregulated and found to demethylate NNMT transcripts in FTO-overexpressing ovarian cancer cells. When NNMT was knocked down or inhibited using 5-amino-1MQ, FTO-induced sensitivity to platinum was rescued, suggesting a novel function of FTO-dependent RNA modifications in regulating the platinum response by targeting the enzyme NNMT [170]. More experimental studies are still needed to evaluate the therapeutic efficacy of these inhibitors, especially in humans.

## 5. Conclusions and Future Prospects

The studies highlighted in this review describe the alterations in expression and the functions of key enzymes involved in NAD^+^ biosynthesis and NAD^+^ consumption in gynecologic cancers. Additionally, the functional role of the enzymes involved in maintaining NAD^+^ homeostasis in controlling biological pathways important for the development and progression of gynecologic cancers has been highlighted. Although these studies describe the importance of developing therapeutic inhibitors for NAD^+^ metabolism to treat gynecologic cancers, therapeutically targeting the NAD^+^ metabolism, unfortunately, has not yielded significant clinical benefits. Hence, new strategies involving combinational treatments may improve the therapeutic outcomes by reducing the dose-limiting toxicity observed with single-agent treatments. There is potential in exploring future research to better understand targeting NAD^+^ metabolism to challenge the persistent issue of therapy resistance. Investigating treatments and biomarkers with high specificity and clinical applicability will help guide the development of precision medicine to patients. Additionally, studies emphasizing the physiological effects of targeting NAD^+^ metabolism and their impact on the tumor microenvironment are required to fully understand the biological outcomes of targeting NAD^+^ metabolism and improve the treatment outcomes for patients.

## Figures and Tables

**Table 1:** Summary of NAD^+^ synthesis pathway enzymes and NAD^+^ consumers in different gynecologic cancers and their influences on cancer cells and the tumor microenvironment.

Enzyme	Cancer Type	Role in Tumors/Tumor Microenvironment	References
NAPRT	Ovarian	Aids in the DNA repair processes by maintaining intracellular NAD^+^ levels	[59]
IDO	Ovarian	Induces immunosuppressive activity through tryptophan metabolism. Increases tumor progression and invasion	[74,79,80]
	Endometrial	Promotes lymph node metastasis Overexpression is correlated with reduced overall survival and disease-free progression	[76,78]
TDO	Ovarian	Causes disease recurrence Correlated with poor survival outcomes	[81]
NAMPT	Ovarian	Influences cancer cell metabolism through NAD^+^ synthesis leading to alterations in DNA repair pathways. Promotes greater cell migration and tumor metastasis.	[82,83]
	Endometrial	Enhances cell and tumor growth through metabolic control.	[84]
NMNAT2	Ovarian	Regulates protein synthesis/proteostasis though PARP16 interaction. Increases cell and decreases overall patient survival	[38]
PARP1	Ovarian	Enhances angiogenesis Overexpression is correlated with poor prognosis, platinum resistance, and overall survival	[85,86,87]
PARP7	Ovarian	Increases cell proliferation through post-translational modifications. Influences migration and motility through modifying a-tubulin.	[37]
PARP16	Ovarian	Increases proteostasis though inhibition of mRNA translation.	[38]
SIRT1	Endometrial	Promotes tumor growth and metastasis	[88]
	Cervical	Promotes tumorigenesis through HPV interaction and promotes disease progression	[89,90]
CD38	Ovarian	Enhances immune cell infiltration	[91]
CD157	Ovarian	Promotes tumor cell proliferation Reduces apoptosis by inhibiting p53	[92]
NNMT	Ovarian	Alters gene expression through regulation of methylation index (SAM:SAH ratio). Regulates metabolism through consuming NAD^+^ and forming 1-methylnicotinamide	[93]

**Table 2:** List of inhibitors targeting NAD^+^ metabolism in gynecologic cancer cell lines, animal models, and clinical trials. In vitro efficacy includes cell growth assays evaluating the efficacy of the treatments in controlling cell growth. In vivo efficacy includes experiments where treating the animals with the inhibitor resulted in a smaller tumor volume or longer survival time. Only data using gynecologic cancer models was included.

Inhibitor	Inhibitor Target(s)	In Vitro Efficacy	In Vivo ** Efficacy	Used in Clinical Trial?	References
Inhibitors of the NAD^+^ biosynthesis pathways					
FK866	NAMPT	Yes	Yes	Yes *	[109,153,154]
FK866 + PARPi	NAMPT PARP	Yes	Yes	No	[155]
FK866 + CD73i	NAMPT CD73	Yes	Yes	No	[156]
FK866 + chemotherapy	NAMPT	Yes	Yes	No	[109]
CHS828	NAMPT	Yes	No	Yes	[109,157,158]
CHS828 + chemotherapy	NAMPT	Yes	Yes	No	[109]
GMX1777	NAMPT	No	No	Yes *	[159]
KPT-9274	NAMPT PAK4	Yes	No	Yes *	[112,160]
2-Hydroxynicotonic acid (2-HNA)	NAPRT	Yes	Yes	No	[68]
Inhibitors of the NAD^+^ consumers					
Olaparib, Rucaparib, Niraparib	PARP	Yes	Yes	Yes	[161,162,163,164,165,166,167,168]
MHY2245	SIRT1	Yes	Yes	No	[169]
5-Amino-1-methylquinolinium (5-amino-1MQ)	NNMT	Yes	Yes	No	[93,170]

* Solid tumors were included in the trial; specific disease sites were not listed in published abstract. ** In vivo studies were performed in mouse models.
